# Biosysthesis of Corn Starch Palmitate by Lipase Novozym 435

**DOI:** 10.3390/ijms13067226

**Published:** 2012-06-12

**Authors:** Jia-Ying Xin, Yan Wang, Tie Liu, Kai Lin, Le Chang, Chun-Gu Xia

**Affiliations:** 1 Key Laboratory for Food Science and Engineering, Harbin University of Commerce, Harbin 150076, China; E-Mails: wangyan_123456@163.com (Y.W.); lylt731@126.com (T.L.); glklkk@126.com (K.L.); katrinachangle@126.com (L.C.); 2 State Key Laboratory for Oxo Synthesis & Selective Oxidation, Lanzhou Institute of Chemical Physics, Chinese Academy of Sciences, Lanzhou 730000, China; E-Mail: cgxia@lzb.ac.cn

**Keywords:** corn starch palmitate, micro-solvent system, solvent-free system, lipase esterification, emulsifying property

## Abstract

Esterification of starch was carried out to expand the usefulness of starch for a myriad of industrial applications. Lipase B from *Candida antarctica*, immobilized on macroporous acrylic resin (Novozym 435), was used for starch esterification in two reaction systems: micro-solvent system and solvent-free system. The esterification of corn starch with palmitic acid in the solvent-free system and micro-solvent system gave a degree of substitution (DS) of 1.04 and 0.0072 respectively. Esterification of corn starch with palmitic acid was confirmed by UV spectroscopy and IR spectroscopy. The results of emulsifying property analysis showed that the starch palmitate with higher DS contributes to the higher emulsifying property (67.6%) and emulsion stability (79.6%) than the native starch (5.3% and 3.9%). Modified starch obtained by esterification that possesses emulsifying properties and has long chain fatty acids, like palmitic acid, has been widely used in the food, pharmaceutical and biomedical applications industries.

## 1. Introduction

Starch is an abundant renewable polysaccharide in nature that is inexpensive, fully biodegradable and widely used in the production of both food and industrial products [1,2]. Chemical modification starch is often required to better suit its properties to specific applications. Many reports exist in literature pertaining to the preparation of starch esters or its components with the ultimate aim of significantly modifying the physical-chemical properties of starches and imparting suitable mechanical characteristics so as to render them more useful as engineering materials than native starch [3,4].

Although the introduction of an ester group into starch is an important chemical modification task [5], it is very difficult to synthesize high substituted starch derivatives, mainly because of the almost impossible proposition of dissolving granular starch in a suitable medium [6,7].

A number of groups have recently reported the use of organic solvents for esterification of starch [8]. Normally, dimethyl sulfoxide (DMSO), dimethyl formamide (DMF) and pyridine are used to dissolve the starch to make it more reactive towards esterification [9]. Some authors [10–14] have reported the preparation of a high degree of starch esters in the presence of organic solvents. Sophisticated experimental techniques and systems of solvents are used to achieve a homogeneous reaction medium for modification of the chosen starch. Unlike chemical esterification modification, an enzymatic one is an environmentally friendly method which occurs under milder conditions. The use of lipase as catalyst for ester production has great potential. In fact, using a biocatalyst eliminates the disadvantages of the chemical process by producing very high purity compounds with fewer or no downstream operations [15].

The aim of this work was to biosynthesize high substituted starch palmitate using lipase Novozym 435 as catalyst. The esterification of starch with palmitic acid was carried out in a solvent-free system and a micro-solvent system respectively. The structure and physicochemical properties of starch palmitate were also studied.

## 2. Materials and Methods

### 2.1. Chemicals and Enzyme

Corn starch was purchased from Harbin Mei Wang Reagent Company, China. The water content was determined by drying the corn starch in a vacuum oven at 50 °C until constant weight was achieved and was 16.2% (w/w). Palmitic acid of analytical grade was purchased from Shanghai Chemical Co., China. DMSO and DMF purchased from Shanghai Chemical Co., China and are of chromatography grade. Novozym 435 (Lipase B from *Candida antarctica* immobilized on macroporous acrylic resin; specific activity: 10,000 U/g) was purchased from Novozymes, Denmark. All the other chemicals are of analytical grade.

### 2.2. Methods

#### 2.2.1. Starch Pretreatment

According to [16] the 9% aqueous solution containing NaOH/Urea at the desired ratio of 2:1 by weight was used as a solvent for starch. The solvent was pre-cooled to below −10 °C. Then the starch sample in the given amount of 5% was added immediately at ambient temperature of below 25 °C. The native starch (NS) was completely dissolved within 5 min by stirring at 3000 r/min and the resultant solution was transparent. The transparent starch solution was neutralized with HCl (15%) until it reached neutrality. Then, starch was precipitated out from the neutral starch solution by adding 50 mL of ethanol drop-wise. After various durations of dropping treatment, the precipitates were washed by successive centrifugations in 95% of ethanol until no HCl remained. Thereafter, they were washed with 100% of ethanol to remove water. The resulting precipitates were vacuum dried at 50 °C for 24 h.

#### 2.2.2. General Procedure for Lipase Esterification

Micro-solvent system: Native corn starch (1 g) was dissolved in 1 mL solvent (DMF/DMSO) and mixed with 1 g palmitic acid and 0.1 g lipase. The homogeneous mixture was incubated in a magnetic stirrer at 55–70 °C, 250 r/min for 3–24 h. The starch palmitate (SP) formed was precipitated by adding 10 mL of 95% alcohol. The removal of unesterified oleic acid from starch palmitate was accomplished by washing again with 100 mL of pure ethanol and then dried in a hot air oven at 75 °C [9].

Solvent-free system: Water activity or a_w_ is an important consideration for biocatalysis in a solvent-free medium. Before the start of the reaction, all the substrates were pre-equilibrated for at least 3 d in sealed containers, enclosed with a molecular sieve to establish fixed water activities (a_w_ < 0.01). The reaction setup for esterification was carried out in 25 mL closed, screw-capped glass vials containing palmitic acid and pretreated starch (PS) or native starch (NS). To conduct the reaction under neat conditions (without solvents), a 5:1 weight ratio of palmitic acid to pretreated starch is needed to provide enough solution volume to dissolve solid starch and to stir the suspended immobilized lipase. The palmitic acid acted as the solvent in the solvent-free system when the reaction temperature was above of its melting point (63–64 °C). The esterification was initiated by adding immobilized lipase (Novozym 435) into each glass vial. Glass vials were placed upright on a magnetic stirrer and incubated at 55–75 °C, 250 r/min for 3–24 h.

#### 2.2.3. Determination of the Degree of Substitution by Methanolysis and GC Analysis

The DS indicates the average number of substitutions per anhydroglucose unit. There are three free hydroxyl groups available for modification per anhydroglucose unit of starch, resulting in a maximum possible DS of 3 [17].

#### 2.2.4. Methanolysis of the Starch Palmitate and GC Analysis of the Methyl Palmitate

A small sample, 30 mg, of starch palmitate dissolved in 1 mL DMSO was mixed with 1 mL of sodium methoxide (0.07 M) in methanol solution. This mixture was then heated (70 °C) under reflux for 40 min, with constant shaking, then cooled, and 1 mL of deionized water and 1 mL of *n*-heptane were added. The mixture was shaken for 1 min and left to settle. The top organic phase contained the methyl ester and could be removed and injected into the GC-FID (Perkin-Elmer Autosystem XL with a CP Simdist capillary column, oven set at 220 °C, the injector at 250 °C and the detector at 260 °C).

#### 2.2.5. Calculation DS of the Starch Palmitate

Once the methyl palmitate was quantified by GC chromatograph, the average mol of acyl groups per anhydroglucose unit was calculated to give the DS of the modified starch. DS was calculated according to the modified method of Kshirsagar [15] as followed (Equation (1)):

(1) $$\text{DS}=\frac{\text{n}\times {\text{M}}_{1}}{{\text{M}}_{0}-\text{n}\times ({\text{M}}_{2}-{\text{M}}_{{\text{H}}_{2}\text{O}})}$$

n: mol of esterifiable palmitic acid, mol;

M_0_: weight of sample, g;

M_1_: molecular weight of anhydrous glucose unit, 162;

M_2_: molecular weight of palmitic acid, 256.42;

M_H_2_O_: molecular weight of H_2_O.

#### 2.2.6. Preparation of Emulsions

Emulsions contained soybean oil (25 mL). The aqueous phase contained 0.5 g starch or starch palmitate. Oil in water emulsions were prepared by first gelatinization of 0.5 g starch or starch palmitate in 25 mL deionized water on a bath of boiling water for 15 min, followed by mixing the oil into the cold starch or starch palmitate paste using a superfine homogenizer FA25 (Fluko Equipment Shanghai Co., China) at 10,000 r/min for 3 min and the emulsion formed. The emulsion was centrifuged at 3000 r/min for 15 min and the height of the emulsion layer was measured, and the total height of liquid. The centrifugation was repeated after 24 h. The emulsifying capacity (EC) and the emulsion stability (ES) were calculated as follows according to the method of Bai [6] (Equations (2) and (3)):

(2) $$\text{EC}=\frac{\text{the height of emulsion layer}}{\text{the total height of liquid}}\times 100\%$$

(3) $$\text{ES}=\frac{\text{the height of emulsion layer after }24\;\text{h}}{\text{the total height of liquid}\;\text{after }24\;\text{h}}\times 100\%$$

#### 2.2.7. Analytical Methods

UV spectrophotometer analysis: The 2% (w/w) native starch or starch palmitate water mixture was prepared hermetically by stirring for 20 min at approximately 95 °C using a magnetic stirrer, and cooled to 25 °C. Deionized water (25 mL) was added into the 50 mL-test tube wherein the gelatinized native starch or starch palmitate (10 mL) was already prepared and neutralized with HCl (2 mol/L) until pH = 3. The emulsion was mixed with 100 mL of deionized water and 0.5 mL of iodine solution (2.0% (w/w) KI and 0.2% (w/w) I_2_ in deionized water) The absorbance (abs) values of the sample and a reference (non-additive sample) were measured at 190–900 nm by UV spectrophotometer (UV-2550, Shimadzu Co.).

FT-IR analysis: FTIR spectra were recorded using a Vector 33 spectrometer (Brucker Company, Munich, Germany). Potassium bromide (KBr) disks were prepared from powdered samples mixed with dry KBr in the ratio of 1:100. The spectra were recorded in a transmittance mode from 4000 to 400 cm^−1^ at a resolution of 4 cm^−1^.

## 3. Results and Discussion

### 3.1. Lipase-Catalyzed Esterification under Micro-Solvent System

**Figure 1:**
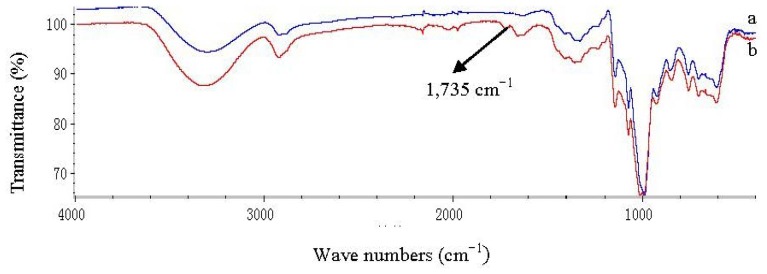
FT-IR spectra of NS (**a**) and SP (**b**) synthesized under micro-solvent system.

**Figure 2:**
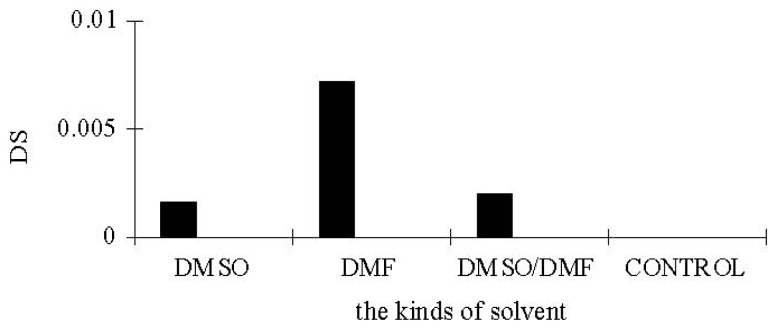
Solvent effect in esterification of starch under micro-solvent system.

**Figure 3:**
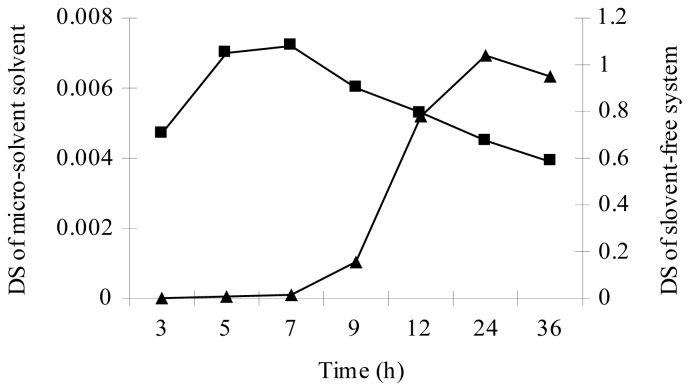
Time effect in DS of starch palmitate synthesized under micro-solvent solvent (■) and solvent-free system (▲).

**Figure 4:**
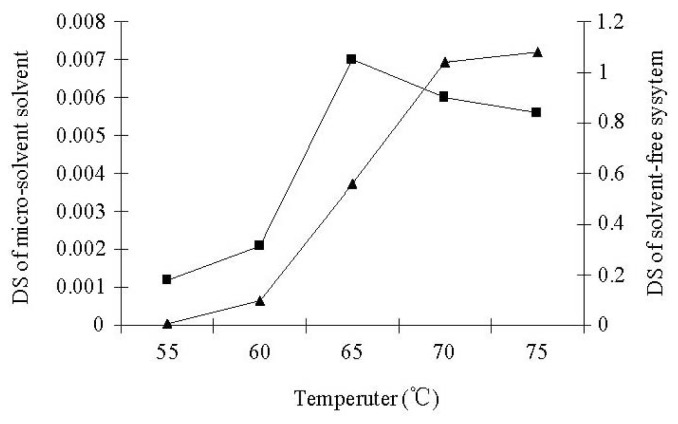
Temperature effect in DS of starch palmitate synthesized under micro-solvent solvent (■) and solvent-free system (▲).

According to the different esterifications of starch with palmitic acid under micro-solvent systems by using DMSO, DMF or the mixture of DMSO and DMF (1:1, v:v) as co-solvent, the micro-solvent system was found to be an effective esterification system for lipase-catalyzed esterifications of starch with palmitic acid. Esterification of native starch with palmitic acid under the micro-solvent system, with DMF as co-solvent, showed an ester peak in the region 1735 cm^−1^ in the IR spectrum (Figure 1). A DS of 0.0072 was obtained in an incubation time of 7 h at 65 °C. Almost no ester peak could be detected in the IR spectrum when using DMSO or the mixture of DMSO and DMF for esterification of starch with palmitic acid. As shown in Figure 2, very common starch solvent DMSO or the mixture of DMSO and DMF gives a worse result when compared to DMF. In all cases, DMF used as a co-solvent gives better reaction effectiveness. The effects of reaction temperature and time on the DS of starch palmitate were investigated. The results (Figures 3,4) showed that the DS of the starch palmitate is temperature and time dependent. The esterification of pretreatment starch with palmitic acid could not be realized under the same conditions regardless of what kind of solvent was used as co-solvent. That may be because the volume expansion of pretreatment starch granules created a situation in which made the micro-solvent could not achieve the role of co-solvent.

### 3.2. Lipase-Catalyzed Esterification under Solvent-Free System

**Figure 5:**
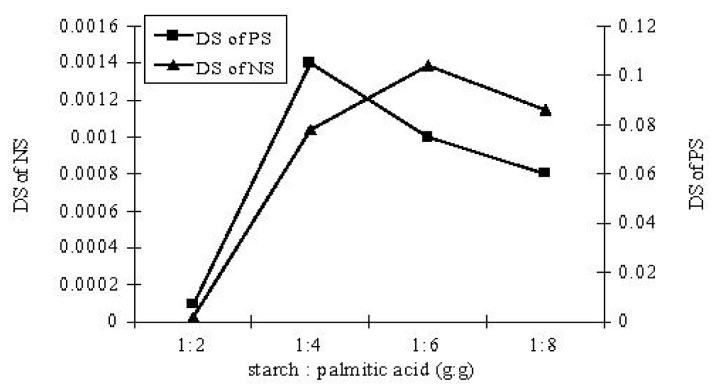
The effect of substrate ratio on the DS of NS and PS.

A number of preliminary experiments were carried out to investigate whether the lipase catalyzed esterification of starch with palmitic acid is indeed feasible in the solvent-free system. The exploratory experiments were performed with lipase Novozym 435 as the catalyst at different substrate ratios and temperatures for 3–24 h. At the end of reaction, the product was soaked in 95% ethanol for 10 min and washed thoroughly with 60 °C methanol to remove un-reacted palmitic acid and dried (50 °C) until constant weight was achieved. The immobilized lipase was removed with 80 screen mesh. Esterification of native starch with palmitic acid under the solvent-free system at reaction conditions (starch:palmitic acid = 1:6, g:g; 70 °C for 24 h) gave an esterification product with a DS of 0.0014. But, the DS of the esterification product was high (to 1.04) after the starch was pretreated using NaOH/Urea/H_2_O (Figure 5). The DS of starch palmitate was very low and could not even be detected when the reaction temperature was at 55 °C or 60 °C. This is mainly because the palmitic acid could not melt at temperature below its melting point, so that the mass transfer between all of the substrates was badly hindered.

### 3.3. UV Spectrum Analysis

**Figure 6:**
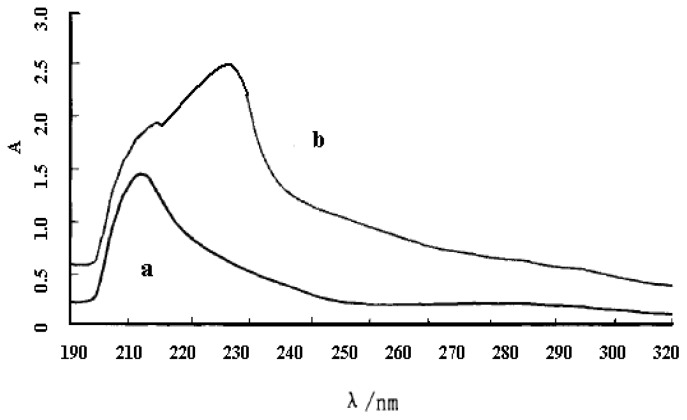
UV spectrum of native starch (**a**) and starch palmitate (**b**) color complex with iodine.

Regardless of the starch solution or the solid starch, a color complex could be generated with iodine, due to the iodine-adsorption action of the starch molecule. The iodine adsorption color reaction would be changed after modification treatment because the size, shape and structure of native starch had been changed. So, the changes in the structure of starch could be understood, according to the difference in the iodine adsorption color reaction between starch palmitate and native starch. Based on the iodine adsorption color reaction, the starch palmitate and native starch were measured by UV-Vis spectrophotometer. The results are as shown in Figure 6.

**Figure 7:**
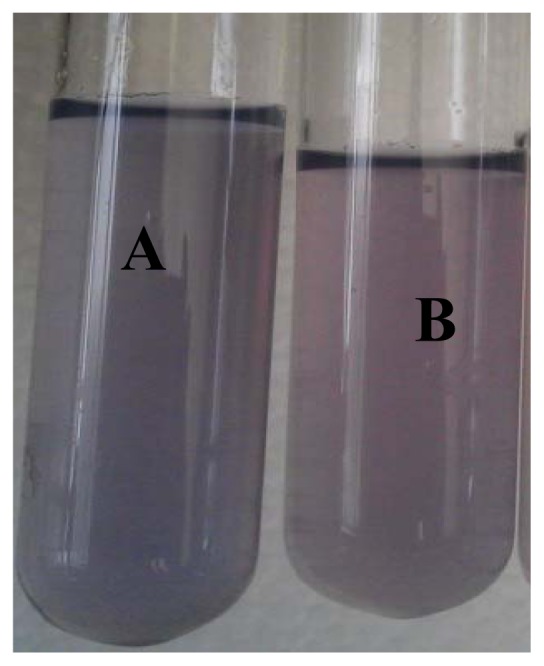
The iodine adsorption action of native starch (**A**) and starch palmicate (**B**).

The iodine adsorption color reaction of native starch presented blue (A), however starch palmitate presented blue purple (B), as shown in Figure 7. The absorbance of starch palmitate (2.495 abs) is lower than native starch (1.413 abs) especially in the maximum absorption wavelength. The maximum absorption wavelength moved to a higher wavelength (from 211 nm to 255 nm) after esterification of starch. The ultraviolet wavelength scan (from 190 nm to 450 nm) results provided preliminary confirmation that the palmitic acid had been introduced into the starch chain.

### 3.4. FT-IR Analysis

**Figure 8:**
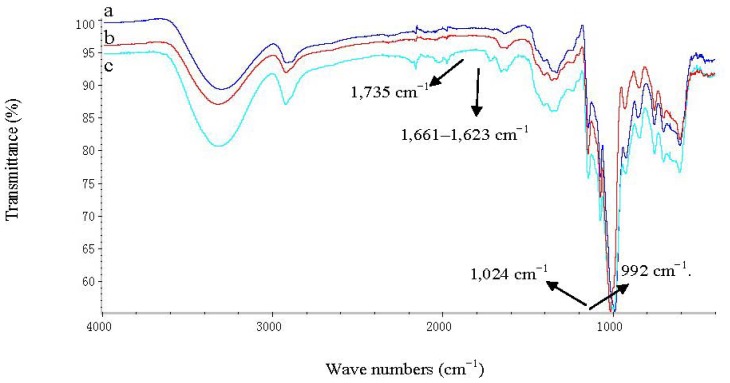
FT-IR spectra of NS (**a**), PS (**b**) and SP (**c**) synthesized under solvent-free system.

In the native starch (NS) spectrum, the characteristic peaks (954–1184 cm^−1^) are attributed to C–OH bond stretching. Another strong broad band, due to hydroxyl bond stretching, appears at 3000–3600 cm^−1^ (Figure 8a,b), which is reduced on starch palmitate (SP), Figure 8c. A characteristic peak C=O bond stretching, present in the starch, occurred at 1661–1623 cm^−1^, which intensified on the pretreatment starch (PS) (Figure 8a,b). That demonstrated the molecular chain of starch had fractured after pretreatment [18]. A strong absorption band at 992 cm^−1^, which appears at 1024 cm^−1^, probably due to the stretching of the C–O–C bond, was present in the spectra of the starch, consistent with the earlier report by Zhao [19]. The red shift of C–O–C bond stretching could weaken the hydrogen bonding. An extremely broad band due to hydrogen bonded hydroxyl groups (O–H) appeared at 3400 cm^−1^ which was attributed to the complex vibrational stretches associated with free, inter- and intra- molecular bound hydroxyl groups that make up the gross structure of starch [20]. The band at 2919 cm^−1^ is characteristic of C–H stretches. These spectra have similar profiles. The differences between NS and PN were the C=O bond stretching and the red shift of C–O–C bond stretching. There was no new peak present.

In comparison with the spectra of the SP, the major change of modified starch is the presence of a carbonyl C–O absorption frequency at 1735 cm^−1^. The strong O–H stretching band at 3400 cm^−1^ in the NS decreased in intensity following esterification of starch with palmitic acid.

### 3.5. Emulsion Capacity and Emulsion Stability

**Figure 9:**
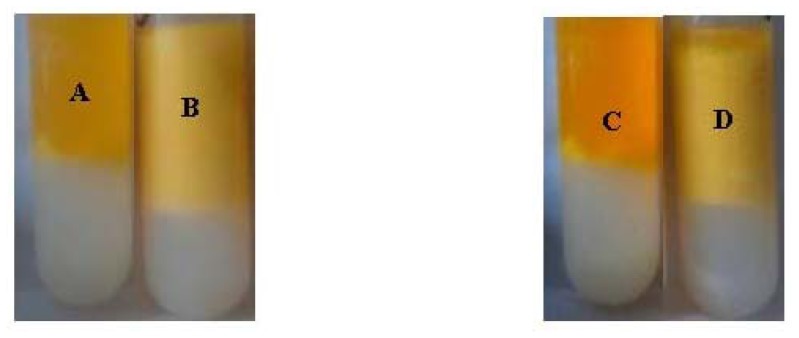
The emulsion of NS (**A**) and SP (**B**) were centrifuged for 15 min; the emulsion of NS (**C**) and SP (**D**) were centrifuged again after storage for 24 h.

Emulsion capacity and emulsion stability were evaluated by visual inspection of the emulsions after different storage times at room temperature. The measurement results of emulsion capacity and emulsion stability of the emulsions are shown in Figure 9. The modified starch obtained the more ideal emulsion capacity (67.6%) and emulsion stability (79.6%) than NS that is only 5.3% and 3.9% respectively. The emulsion capacity and emulsion stability of starch palmitate did not change significantly during the storage period.

## 4. Conclusions

Starch palmitate was successfully synthesized through esterification of starch with palmitic acid catalyzed by Novozym 435 lipase. Compared with corn starch, the carbonyl band of starch ester produced by esterification had been detected by IR spectrum. In a micro-solvent esterification system, esterification of native starch with palmitic acid was carried out at 10% (w/w) lipase and 65 °C for 7 h, a maximum DS of 0.0072 was obtained. As for a solvent-free system, esterification of corn starch with palmitic acid was carried out at 5% (w/w) lipase and 70 °C for 24 h, a maximum DS of 1.04 was obtained. Compared to the micro-solvent system, solvent-free system esterification is a more suitable method to produce high DS long chain fatty acid starch ester. However, a micro-solvent system is a more economical way for obtain low DS long chain fatty acid starch esters. The introduction of palmitic acid into starch renders starch more hydrophobic which would lead to the starch possessing excellent emulsion capacity and emulsion stability.
